# Do nutrition knowledge and health literacy influence food consumption patterns in Saudi youth aged 18 to 25? An analytical assessment using cross-sectional convenience sample

**DOI:** 10.3389/fpubh.2026.1806616

**Published:** 2026-04-09

**Authors:** Mohannad A. Alzain

**Affiliations:** 1 Department of Family Medicine, Faculty of Medicine, King Abdul Aziz University, Jeddah, Saudi Arabia; 2 Department of Family Medicine, King Abdulaziz University Hospital, King Abdulaziz University, Jeddah, Saudi Arabia; 3 Family Medicine and Chronic Diseases Research Unit, King Fahd Medical Research Center, King Abdul Aziz University, Jeddah, Saudi Arabia

**Keywords:** dietary behaviors, food consumption patterns, health literacy, nutrition knowledge, Saudi Arabia, young adults

## Abstract

**Objective:**

Nutrition knowledge and health literacy are critical foundations for healthy dietary behaviors. However, their combined influence on food consumption patterns in Saudi young adults remains underexplored. This research investigated the levels and predictors of nutrition knowledge and health literacy, as well as their impact on food consumption patterns among a group of Saudi Arabian young people.

**Methods:**

This was an online survey (structured, self-administered questionnaire) using a large convenience sample of students from different universities in Saudi Arabia. Nutrition knowledge, health literacy, and food consumption patterns were evaluated using the validated CoNKS, the HLS-EU-Q16, and a regionally adapted 10-item food group checklist, respectively. Regression models were fitted to evaluate the predictors of nutrition knowledge and health literacy, and their associations with food consumption frequency.

**Results:**

The sample comprised 827 participants, aged 18 to 25. The prevalence of unsatisfactory nutrition knowledge was 70.5% and limited health literacy was 58.3%. Significant factors associated with unsatisfactory nutrition knowledge included non-health science major, physical inactivity, no prior nutrition course, night eating syndrome, and limited health literacy. Key factors associated with limited health literacy included age (18–20 years), non-health science major, having an illiterate mother, and unsatisfactory nutrition knowledge. Furthermore, satisfactory nutrition knowledge and sufficient health literacy were independently associated with healthier food consumption patterns. Specifically, they were linked to higher odds of consuming fruits and vegetables (nutrition knowledge: OR = 1.76; health literacy: OR = 2.71) and meat, fish, and eggs (nutrition knowledge: OR = 2.67). Unsatisfactory nutrition knowledge was related to high-calorie foods intake (OR = 3.70).

**Conclusion:**

Both nutrition knowledge and health literacy are significant, modifiable factors associated with food consumption patterns among study participants. Integrated educational interventions targeting both competencies are recommended to improve dietary behaviors in this population.

## 1 Introduction

Nutrition knowledge, health literacy, and dietary diversity are among the important parameters of an individual’s nutritional behaviors and health status (1, 2). Globally, the nutrition transition towards ultra-processed foods has redefined eating patterns, often at the expense of meal regularity and dietary quality (3). In Saudi Arabia, risky and undesirable nutritional behaviors such as intake of high-calorie processed food items have been observed predominantly, which is responsible for obesity, metabolic disorders and non-communicable diseases (NCDs) (4–6). NCDs are the top contributors of death in Saudi Arabia (7), affecting roughly three-quarters of deaths (73%, in 2020) (8). Within the Gulf Cooperation Council, Saudi Arabia bears the largest share of this burden, contributing to 45% of NCD-related deaths and 60% of the associated economic losses (9). A significant share of this mortality is directly attributable to modifiable dietary risks, including elevated salt and sugar intake and inadequate fruit and vegetable consumption (10, 11).

Addressing poor dietary patterns requires adequate nutrition knowledge, a foundational and modifiable determinant of diet quality and disease prevention. However, a widespread deficiency exists. For instance, a multi-national Middle Eastern study found that about 73% of participants’ nutrition knowledge was inadequate (12), highlighting an urgent need for educational interventions. Adequate nutrition knowledge is a key determinant of dietary behavior, as it supports healthier food choices, encourages evidence-based practices such as appropriate use of nutrition labels, and promotes higher consumption of vegetables and lean protein sources. Collectively, these behaviors are associated with a lower risk of NCDs (13, 14). Therefore, assessing nutrition knowledge is critical for identifying gaps and designing effective strategies to enhance good nutrition and dietary behaviors (15, 16).

Health literacy (i.e., getting capacity, comprehend, and application of health information) is essential in disease prevention and control (1). In Saudi Arabia, health literacy is significantly associated with various factors, including educational level, age, and academic discipline, with non-healthcare students and those with lower educational backgrounds demonstrating higher vulnerability to limited health literacy (17–20). International studies have shown that poor health outcomes and a low healthy eating index are linked to low or inadequate health literacy (21, 22). Research on the link between health literacy and food consumption patterns in Saudi Arabian youth is notably scarce, despite evidence suggesting gaps in nutrition understanding.

As a whole, university students (e.g., young adults) fall into crucial age groups for making lifelong health behaviors. Saudi-based research indicates they are prone to leading unhealthy lives characterized by poor dietary habits and a sedentary lifestyle (23). This population (Saudi university students) is increasingly exposed to lifestyle risks within the academic environment, including stress and the widespread availability of energy-dense refined foods. This setting has been linked to a dietary transition away from traditional foods towards less healthy patterns, such as consuming more processed and fast foods (24, 25). As diversity in foods is one of the core components of the overall quality of diet (26), such shifts raise significant public health concerns. While poor nutrition knowledge and unhealthy food patterns have been separately noted in this population, these factors are seldom examined alongside health literacy. An integrated understanding is vital, as informed dietary choices depend on both knowledge and usage ability. The interrelationship between nutrition knowledge, health literacy, and food consumption patterns warrants deeper exploration, particularly given the potential for modifiable behaviors to mitigate NCD risks. So, this research aims to concurrently study nutrition knowledge, health literacy, and food consumption patterns among young adults in Saudi Arabia.

Key research questions as a guide:

*Primary research question:* How are nutrition knowledge and health literacy associated with food consumption patterns?

*Secondary research question:* (i) What are the observed prevalence rates of nutrition knowledge (satisfactory and unsatisfactory), health literacy (sufficient and limited), and food consumption patterns among the participants? (ii) Which variables predict unsatisfactory nutrition knowledge and limited health literacy?

## 2 Methods

### 2.1 Ethics and research design

This cross-sectional research secured the study clearance from the ethics authority of University of Jeddah (approval number: UJ-REC-181). The entire research process was conducted following the relevant ethical guidelines and regulatory standards. Participation in this research was optional and Informed consent was taken. All personal data were treated with strict confidentiality.

### 2.2 Participants and eligibility criteria

This study targeted Saudi students from multiple universities. University is the best and feasible source for recruiting young adults, which is why this study enrolled participants from this setting. Eligible participants were Saudi citizens currently enrolled in a university and aged between 18 and 30 years. However, this study was able to recruit participants from the age range of 18–25 years. Individuals were excluded if they had a clinically diagnosed eating disorder or any severe psychiatric diagnosis (e.g., depressive and bipolar disorder, schizophrenia).

### 2.3 Sample size and sampling

The minimum required sample (n) was estimated for the study’s three co-primary outcomes using the single population proportion formula: $n=\frac{z^{2}\times p\times (1-p)}{d^{2}}$

Where *Z =* 1.96 (with 95% confidence), *p* = the anticipated prevalence of the outcome, and *d* = 0.05 (error level). Anticipated prevalence rates were sourced from previous studies in Saudi Arabian populations: unsatisfactory nutrition knowledge (51.3%) (27), inadequate health literacy (54.4%) (18), and high fast food consumption (86.7%) (28). Since the sample size requirement is maximized for proportions closest to 50%, the calculation was based on unsatisfactory nutrition knowledge (*p* = 0.513). This provided an initial sample of 384. After adjusting for an estimated 15% non-response rate, the adjusted target was 452 participants. The final analyzed sample comprised 827 young adults, which substantially exceeded this target, providing greater precision and statistical power for all planned analyses

A non-probability convenience sampling approach was followed. Students from various universities were invited to complete a structured online questionnaire (Google Forms). Through online applications and platforms for social communication, the survey link was disseminated by the data surveyors’ personal correspondence. The email and social media invitations contained a short description of the research purpose and other necessary details. The survey remained active for one and a half months to ensure adequate time for participant response. The last 2 months of 2025 were the survey phase.

### 2.4 Study variables

**Figure 1:**
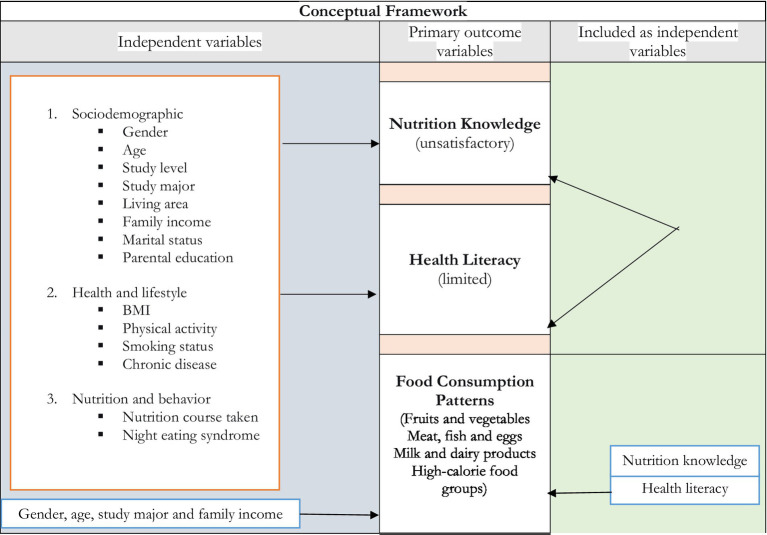
Conceptual framework mapping independent variables to the study outcomes.

The guiding scheme for this investigation is shown in Figure 1, which maps the independent variables tested against each of the three simultaneously assessed outcome variables: (i) nutrition knowledge, (ii) health literacy, and (iii) food consumption patterns. The content and measurement procedures for each variable are described below.

#### 2.4.1 Outcome variables

*Nutrition knowledge:* A validated consumer nutrition knowledge scale (CoNKS) was used to measure nutrition knowledge (29). This 20-item questionnaire assesses both declarative nutrition knowledge (e.g., calorie and nutrient content, and food composition) and procedural knowledge (e.g., health benefits of food groups) using ‘yes’, ‘no’, or ‘do not know’ responses. Scores range from 0 to 20, with one value given for each accurate response. In line with the previous application, a point range of 16 to 20 was classified as satisfactory nutrition knowledge, and a point of <16 as unsatisfactory knowledge (12). This tool has been previously used among the general population in Arab nations like Saudi Arabia, where the tool demonstrated a good level of reliability coefficient (Cronbach’s alpha = 0.797) (12). In the present study, this scale also showed a good level of reliability (Cronbach’s alpha = 0.713).

*Health literacy:* Health literacy was assessed using the HLS-EU-Q16, a questionnaire derived from the European Health Literacy Survey (30, 31). Its validation among Arabic-speaking populations in other regions supports its applicability for the present study in Saudi Arabia (30, 32). This 16-item tool measures an individual’s perceived ability to access, understand, appraise, and apply health information across three domains: healthcare (items 1–7), disease prevention (items 8–12), and health promotion (items 13–16). Responses of ‘very easy’ or ‘easy’ were rated as 1, while ‘difficult’ or ‘very difficult’ were rated as zero. A final score was computed for each participant (range 0–16) and categorized into three levels: ‘sufficient’ (score ≥13), ‘problematic’ (score 9–12), or ‘inadequate’ (score ≤8) (30). For analysis, the ‘problematic’ and ‘inadequate’ categories were combined to represent ‘limited health literacy’.

*Food consumption patterns:* Food consumption pattern was evaluated by a 10-item food group checklist (24-h recall), adapted from a previous Saudi-based study on regional food consumption patterns (33). The checklist contained the following food groups: (1) Cereal and its products, (2) Starchy roots, (3) Legumes, (4) Vegetables and fruits, (5) Meat, fish, and eggs, (6) Milk and dairy products, (7) Polysaccharides, canned food, and juices, (8) Fats and oils, (9) Beverages, and (10) Spices, appetizers, and fast foods. Responses were recorded as ‘yes’ or ‘no’. For analytical purposes, a “high-calorie food group” variable was created from four energy-dense or processed groups (7 to 10 groups). The number of high-calorie food groups consumed by each participant was summed, producing a score ranging from 0 to 4. This score was then used to categorize individuals into three levels of high-calorie food consumption: Low (consuming 0–1 items), Moderate (consuming 2 items), or High (consuming 3–4 items). This categorization was chosen for observing associations with nutrition knowledge and health literacy, as consuming multiple high-calorie food groups may better reflect dietary habits than any single group alone.

#### 2.4.2 Independent variables and covariates

Our study incorporated various sociodemographic, academic, and health-related covariates. These included gender, age, study level and major, marital status, living area, and monthly family income. Parental education levels were also considered. Health and lifestyle factors comprised body mass index category, physical activity level, smoking status, the presence of chronic disease, and whether the participant had taken a nutrition-related course. Additionally, night eating syndrome was screened using night eating questionnaire (34).

### 2.5 Data analysis

The chi-square test assessed the initial relationships between the outcomes and predictor variables. Separate adjusted binary logistic regression approaches were utilized to reveal the factors linked to unsatisfactory nutrition knowledge and limited health literacy. The influence of nutrition knowledge and health literacy on food consumption patterns was evaluated through binary logistic regression (adjusted) for three food groups: (1) fruits and vegetables (consumed vs. not consumed), (2) meat, fish and eggs (consumed vs. not consumed) and (3) milk and dairy products (consumed vs. not consumed). Additionally, multinomial logistic regression was employed to examine how nutrition knowledge and health literacy are associated with high and medium consumption of high-calorie food groups, with low consumption as the reference. These food consumption patterns models were adjusted for potential confounders, including gender, age, study major, and family income. Multicollinearity among independent variables was examined via the variance inflation factor, which indicated no collinearity. All adjusted regression models satisfied the criteria of HL test (Hosmer-Lemeshow). STATA software (v.16) was used to perform the analysis (significance level: *p* < 0.05).

## 3 Results

### 3.1 Sample characteristics

The participants (*N* = 827) were almost evenly distributed by gender (50.3% male) and the majority (70.0%) were between 21 to 25 years. Sample details are outlined in Table 1.

**Table 1:** Characteristics of the study participants (*n* = 827).

Variables	Level	Frequency and percent
Gender	Male	416 [50.3%]
	Female	411 [49.7%]
Age (years)	18–20	248 [30.0%]
	21–25	579 [70.0%]
Study level	Undergraduate	640 [77.4%]
	Post-graduate	187 [22.6%]
Study major	Health science	443 [53.6%]
	Non-health science	384 [46.4%]
Living area	Own house	412 [49.8%]
	Rental house	200 [24.2%]
	Dormitory	215 [26.0%]
Monthly family income	<5,000 SAR	105 [12.7%]
	5,000–<10,000 SAR	217 [26.2%]
	10,000–15,000 SAR	323 [39.1%]
	>15,000	182 [22.0%]
Marital status	Unmarried	769 [93.0%]
	Married	58 [7.0%]
Mother education	Illiterate	98 [11.9%]
	Primary education	346 [41.8%]
	Secondary and high education	383 [46.3%]
Father education	Illiterate	80 [9.7%]
	Primary education	253 [30.6%]
	Secondary and high education	494 [59.7%]
BMI	Underweight	122 [14.8%]
	Normal	495 [59.9%]
	Overweight or obese	210 [25.4%]
Physical activity	Inactive	295 [35.7%]
	Moderate activity	339 [41.0%]
	Regular physical activity	193 [23.3%]
Smoking	Yes	91 [11.0%]
	No	736 [89.0%]
Having chronic disease	Yes	218 [26.4%]
	No	609 [73.6%]
Took nutrition-related course	Yes	408 [49.3%]
	No	419 [50.7%]
Night eating syndrome	Yes	125 [15.1%]
	No	702 [84.9%]

### 3.2 Nutrition knowledge: prevalence and associated factors

**Figure 2:**
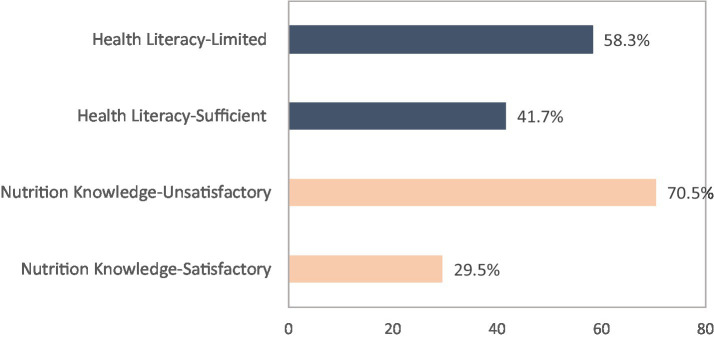
Prevalence of nutrition knowledge and health literacy among the 827 study participants.

Nearly three-quarters (70.5%) of the respondents had unsatisfactory nutrition knowledge and 29.5% had satisfactory nutrition knowledge (Figure 2). Initial significant relation was ascertained by chi-square analysis, showing nutrition knowledge was significantly related to participants’ study major (*p* = 0.002), family income (*p* = 0.027), parent education (*p* < 0.05), smoking habit (*p* < 0.001), taking nutritional course (*p* = 0.001), night eating syndrome (*p* < 0.001) and health literacy (*p* < 0.001; Table 2).

**Table 2:** Distribution of nutrition knowledge and health literacy by independent variables.

Variables	Nutrition knowledge			Health literacy		
	Unsatisfactory	Satisfactory	*p*	Limited	Sufficient	*p*
Gender						
Male	283 (68.0)	133 (32.0)	0.118	222 (53.4)	194 (46.6)	**0.004**
Female	300 (73.0)	111 (27.0)		260 (63.3)	151 (36.7)	
Age (years)						
18–20	174 (70.2)	74 (29.8)	0.890	168 (67.7)	80 (32.3)	**0.000**
21–25	409 (70.6)	170 (29.4)		314 (54.2)	265 (45.8)	
Study level						
Undergraduate	446 (69.7)	194 (30.3)	0.346	359 (56.1)	281 (43.9)	**0.018**
Post-graduate	137 (73.3)	50 (26.7)		123 (65.8)	64 (34.2)	
Study major						
Health science	206 (46.50)	237 (53.50)	**0.002**	155 (35.0)	288 (65.0)	**0.000**
Non-health science	266 (69.3)	118 (30.7)		194 (50.5)	190 (49.5)	
Living area						
Own house	285 (69.2)	127 (30.8)	0.052	224 (54.4)	188 (45.6)	0.068
Rental house	133 (66.5)	67 (33.5)		122 (61.0)	78 (39.0)	
Dormitory	165 (76.7)	50 (23.3)		136 (63.3)	79 (36.7)	
Monthly family income						
<5,000 SAR	61 (58.1)	44 (41.9)	**0.027**	54 (51.4)	51 (48.6)	0.308
5,000–<10,000 SAR	160 (73.7)	57 (26.3)		124 (57.1)	93 (42.9)	
10,000–15,000 SAR	231 (71.5)	92 (28.5)		190 (58.8)	133 (41.2)	
>15,000	131 (72.0)	51 (28.0)		114 (62.6)	68 (37.4)	
Marital status						
Unmarried	538 (70.0)	231 (30.0)	0.219	449 (58.9)	320 (41.6)	0.824
Married	43 (77.6%)	13 (22.4)		33 (56.9)	25 (43.1)	
Mother education						
Illiterate	77 (78.6)	21 (21.4)	**0.037**	72 (73.5)	26 (26.5)	**0.005**
Primary education	251 (72.5)	95 (27.5)		194 (56.1)	152 (43.9)	
Secondary and high education	255 (66.6)	128 (33.4)		216 (56.4)	167 (43.6)	
Father education						
Illiterate	36 (45.0)	44 (55.0)		28 (35.0)	52 (65.0)	**0.000**
Primary education	210 (83.0)	43 (17.0)	**0.000**	162 (64.0)	91 (36.0)	
Secondary and high education	337 (68.2)	157 (31.8)		292 (59.1)	202 (40.9)	
BMI						
Underweight	89 (73.0)	33 (27.0)	0.795	64 (52.5)	58 (47.5)	0.058
Normal	348 (70.3)	147 (29.7)		305 (61.6)	190 (38.4)	
Overweight or obese	146 (69.5)	64 (30.5)		113 (53.8)	97 (46.2)	
Physical activity						
Inactive	218 (73.9)	77 (26.1)	0.217	180 (61.0)	115 (39.0)	0.400
Moderate activity	229 (67.6)	110 (32.4)		196 (57.8)	143 (42.2)	
Regular physical activity	136 (70.5)	57 (29.5)		106 (54.9)	87 (45.1)	
Smoking						
Yes	45 (49.5)	46 (50.5)	**0.000**	39 (42.9)	52 (57.1)	**0.002**
No	538 (73.1)	198 (26.9)		443 (60.2)	293 (39.8)	
Having chronic disease						
Yes	153 (70.2)	65 (29.8)	0.906	133 (61.0)	85 (39.0)	0.341
No	430 (70.6)	179 (29.4)		349 (57.3)	260 (42.7)	
Took nutrition related course						
Yes	266 (65.2)	142 (34.8)	**0.001**	218 (53.4)	190 (46.6)	**0.005**
No	317 (75.7)	102 (24.3)		264 (63.0)	155 (37.0)	
Night eating syndrome						
Yes	106 (84.8)	19 (15.2)	**0.000**	80 (64.0)	45 (36.0)	0.159
No	477 (67.9)	225 (32.1)		402 (57.3)	300 (42.7)	
Nutrition knowledge	Not Included					
Satisfactory				71 (29.1)	173 (70.9)	**0.000**
Unsatisfactory				411 (70.5)	172 (29.5)	
Health literacy				Not included		
Sufficient	172 (49.9)	173 (50.1)	**0.000**			
Limited	411 (85.3)	71 (14.7)				

Bolded values indicate significant result.

The adjusted logistic regression model identified several significant factors associated with unsatisfactory nutrition knowledge (Table 3). Participants with a non-health science major had 2.55 times higher odds of having unsatisfactory knowledge than those in health science majors (OR = 2.55, 95%CI = 1.45–4.49, *p* = 0.001). Respondents who were involved in moderate physical activity had 46% lower odds of having unsatisfactory nutrition knowledge than those who were inactive (OR = 0.54, 95%CI = 0.36–0.81, *p* = 0.003). Respondents who had taken a nutrition-related course were less prone to demonstrate unsatisfactory nutrition knowledge than those without such course (OR = 0.28, 95%CI = 0.15–0.49, *p* < 0.001). Moreover, night eating syndrome (OR = 4.35, 95%CI = 2.28–8.31, *p* < 0.001) and limited health literacy (OR = 6.35, 95%CI = 4.37–9.22, *p* < 0.001) were related with increased odds of having unsatisfactory nutrition knowledge.

**Table 3:** Predictors of unsatisfactory nutrition knowledge among a sample of young adults in Saudi Arabia (adjusted binary regression model).

Variables	OR	p	95% CI
Gender			
Male (vs. female)	1.04	0.835	0.72–1.52
Age (years)			
21–25 (vs. 18–20)	1.07	0.746	0.71–1.63
Study level			
Post-graduate (vs. undergraduate)	0.71	0.175	0.43–1.16
Study major			
Non-health science (vs. health science)	2.55	**0.001**	1.45–4.49
Living area			
Rental house (vs. own house)	0.67	0.072	0.44–1.04
Dormitory (vs. own house)	1.44	0.134	0.89–2.30
Monthly family income (SAR)			
5,000 to <10,000 (vs. <5,000)	2.06	0.124	0.86–3.70
10,000 to 15,000 (vs. <5,000)	1.86	0.091	0.91–3.19
>15,000 (vs. <5,000)	1.49	0.183	0.82–2.71
Marital status			
Married (vs. unmarried)	1.83	0.106	0.88–3.82
Mother education			
Illiterate (vs. secondary and high education)	1.27	0.458	0.67–2.38
Primary education (vs. secondary and high education)	1.43	0.096	0.94–2.18
Father education			
Illiterate (vs. secondary and high education)	0.27	0.098	0.15–1.51
Primary education (vs. secondary and high education)	1.66	0.127	0.96–2.59
BMI			
Underweight (vs. normal)	1.42	0.191	0.84–2.39
Overweight or obese (vs. normal)	1.01	0.966	0.66–1.55
Physical activity			
Moderate activity (vs. inactive)	0.54	**0.003**	0.36–0.81
Regular physical activity (vs. inactive)	1.04	0.889	0.63–1.69
Smoking			
No (vs. yes)	1.76	0.061	0.97–3.19
Having chronic disease			
No (vs. yes)	1.07	0.713	0.722–1.61
Took nutrition related course			
Yes (vs. no)	0.28	**0.000**	0.15–0.49
Night eating syndrome			
Yes (vs. no)	4.35	**0.000**	2.28–8.31
Health literacy			
Limited (vs. sufficient)	6.35	**0.000**	4.37–9.22

OR, odds ratio, CI, confidence interval. Bolded values indicate significant result. Hosmer-Lemeshow test statistics: chi-square = 12.31, *p* = 0.163.

### 3.3 Health literacy: level and associated factors

Over half of the sample (58.3%) had limited health literacy and 41.7% had sufficient health literacy (Figure 2). An initial association found health literacy significantly associated with the following variables: sex (*p* = 0.004), age (*p* = 0.001), study major (*p* = 0.000), study level (*p* = 0.018), parent education (*p* < 0.05), smoking status (*p* = 0.002), taking nutritional course (*p* = 0.005) and nutrition knowledge (*p* < 0.001).

Table 4 provided the output of the adjusted binary regression model identifying the factors associated with limited health literacy. Participants aged 21–25 years were less likely to possess limited health literacy than the comparison age-group (OR = 0.53, 95%CI = 0.36–0.77, *p* = 0.001). The odds of limited health literacy were higher for non-health science majors compared to health science majors (OR = 2.56, 95%CI = 1.58–4.14, *p* < 0.001). Participants whose mothers were illiterate had an increased probability of having limited health literacy relative to those whose mothers had a secondary or higher education (OR = 1.84, 95% CI = 1.03–3.28, *p* = 0.039). Unsatisfactory nutrition knowledge was associated with an elevated probability of limited health literacy (OR = 6.23, 95% CI = 4.29–9.05, *p* < 0.001).

**Table 4:** Predictors of limited health literacy among a sample of young adults in Saudi Arabia (adjusted binary regression model).

Variables	OR	P value	95% CI
Gender			
Male (vs. female)	0.74	0.085	0.53–1.04
Age (years)			
21–25 (vs. 18–20)	0.53	**0.001**	0.36–0.77
Study level			
Post-graduate (vs. undergraduate)	1.18	0.466	0.76–1.84
Study major			
Non-health science (vs. health science)	2.56	**0.000**	1.58–4.14
Living area			
Rental house (vs. own house)	1.41	0.089	0.95–2.09
Dormitory (vs. own house)	1.39	0.152	0.89–2.03
Monthly family income (SAR)			
5,000 to <10,000 (vs. <5,000)	1.12	0.674	0.65–1.95
10,000 to 15,000 (vs. <5,000)	1.23	0.660	0.67–1.88
>15,000 (vs. <5,000)	1.33	0.316	0.76–2.33
Marital status			
Married (vs. unmarried)	0.68	0.219	0.37–1.26
Mother education			
Illiterate (vs. secondary and high education)	1.84	**0.039**	1.03–3.28
Primary education (vs. secondary and high education)	1.05	0.779	0.73–1.51
Father education			
Illiterate (vs. secondary and high education)	0.44	0.097	0.23–1.02
Primary education (vs. secondary and high education)	0.88	0.498	0.61–1.27
BMI			
Underweight (vs. normal)	0.56	0.079	0.33–1.05
Overweight or obese (vs. normal)	0.64	0.082	0.42–1.04
Physical activity			
Moderate activity (vs. inactive)	0.88	0.489	0.61–1.27
Regular physical activity (vs. inactive)	0.76	0.213	0.49–1.17
Smoking			
Yes (vs. no)	0.59	0.085	0.33–1.07
Having chronic disease			
Yes (vs. no)	1.24	0.237	0.87–1.89
Took nutrition related course			
Yes (vs. NO)	1.64	0.053	0.99–2.71
Night eating syndrome			
Yes (vs. no)	0.82	0.420	0.51–1.33
Nutrition knowledge			
Unsatisfactory (vs. satisfactory)	6.23	**0.000**	4.29–9.05

OR, odds ratio; CI, confidence interval. Bolded values indicate significant result. Hosmer-Lemeshow test statistics: chi-square = 10.21, *p*-value = 0.193.

### 3.4 Food consumption patterns: role of nutrition knowledge and health literacy

**Figure 3:**
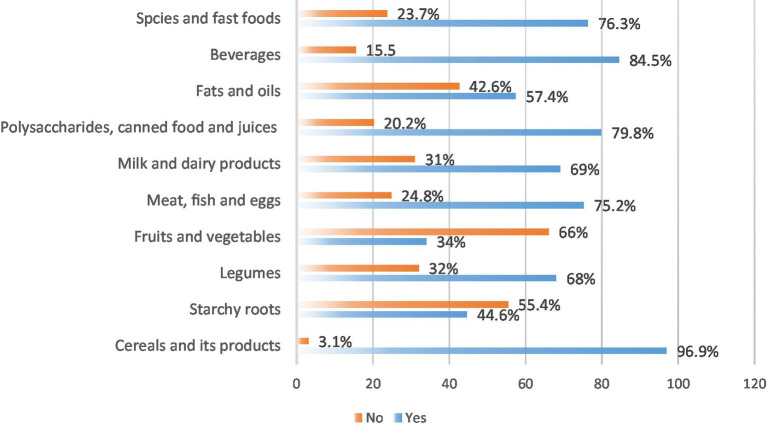
Frequency of food group consumption in the 24-h period before the survey among study participants (*N* = 827).

Consumption of several healthy or core food groups was high, with nearly all participants (96.9%) consuming cereals and their products. A majority also reported consuming meat, fish, and eggs (75.2%), legumes (68.0%), and milk and dairy products (69.0%). However, consumption of vegetables and fruits was comparatively low (34.0%). Consumption of less healthy or discretionary items was also prevalent. A large majority consumed beverages (84.5%), items categorized as polysaccharides, canned food, and juices (79.8%), as well as spices, and fast foods (76.3%) (Figure 3).

The regression models examining the relation among nutrition knowledge, health literacy, and food group consumption (Table 5). Compared to the respective counterparts, both satisfactory nutrition knowledge (OR = 1.76, 95%CI = 1.19–2.58, *p* = 0.004) and sufficient health literacy (OR = 2.71, 95%CI = 2.59–9.80, *p* < 0.001) were correlated with higher frequency of fruits and vegetables intake. Moreover, satisfactory nutrition knowledge was significantly associated with higher consumption of meat, fish, and eggs (OR = 2.67, 95%CI: 1.82–3.92, *p* < 0.001). Unsatisfactory nutrition knowledge was related with higher odds of high consumption of high-calorie foods compared to satisfactory knowledge (OR = 3.70, 95% CI = 1.099–5.87, *p* < 0.001).

**Table 5:** Association of nutrition knowledge and health literacy with the consumption of different food groups among a sample of young adults in Saudi Arabia.

Independent variables	Different food groups			
	OR	95% CI	*p*	
Nutrition knowledge				Fruits and vegetables
Satisfactory	1.76	[1.19–2.58]	**0.004**	
Unsatisfactory	Reference category			
Health literacy				
Sufficient	2.71	[2.19–7.80]	**0.000**	
Limited	Reference category			
Nutrition knowledge				Meat, fish and eggs
Satisfactory	2.67	[1.82–3.92]	**0.000**	
Unsatisfactory	Reference category			
Health literacy				
Sufficient	1.17	[0.84–1.63]	0.351	
Limited	Reference category			
Nutrition knowledge				Milk and dairy products
Satisfactory	1.09	[0.76–1.57]	0.623	
Unsatisfactory	Reference category			
Health literacy				
Sufficient	0.74	[0.53–1.03]	0.074	
Limited	Reference category			

Independent variables	Consumption frequency of high-caloric-energy food groups	
	Medium (vs. low)	High (vs. low)
Unsatisfactory nutrition knowledge	OR = 0.79 95%CI = [0.37–1.67] *p* = 0.540	OR = 3.7 95%CI = [1.099–5.87] *p* = **0.00**
Limited health literacy	OR = 1.84 95%CI = [0.94–3.62] *p* = 0.07	OR = 1.68 95%CI = [0.88–3.23] *p* = 0.117

OR, odds ratio, CI, confidence interval. Each model was adjusted for gender, age, study major, and family income. Bolded values indicate significant result.

## 4 Discussion

### 4.1 Prevalence and correlates of nutrition knowledge

In the present study, almost three-fourths (70.5%) of Saudi young population were found to have unsatisfactory nutrition knowledge. This high prevalence is consistent with a recent multi-national Arab study among general people, which reported 73.1% unsatisfactory nutrition knowledge (12). However, another study among Saudi young adults shows 51.3% had unsatisfactory knowledge (27). A further study among young Arabs aged 18 to 25 years across several countries revealed that only 43.38% had a satisfactory level of knowledge about healthy diets (35). The variability in measurement tools or sample characteristics is a reason for the fluctuation in percentage. Therefore, setting up programs for organized, evidence-based nutrition education and administering them across the country are public health priorities. To contribute to the health and wellness goals of Saudi Vision 2030, these initiatives should be integrated into educational curricula, public health campaigns, and digital platforms to effectively improve nutrition knowledge among youth.

Our findings demonstrate that both formal academic discipline and targeted coursework are critical determinants of nutrition knowledge. The significantly higher odds of unsatisfactory knowledge among non-health science majors underscore a fundamental disciplinary gap, a finding consistent with prior cross-sectional research. For instance, a study by (36) reported significantly higher median nutrition knowledge scores among UK healthcare students compared to their non-healthcare peers (66·0 vs. 62·0, *p* < 0.05), confirming this established disparity. This pattern, where health science students possess superior health awareness (37), is largely attributable to the integrated nature of their core curricula (38, 39). However, this gap is modifiable. Our second finding shows that taking a dedicated nutrition course significantly reduced the odds of poor knowledge, irrespective of a student’s major. This indicates the core issue is a lack of specific educational exposure, not the discipline itself. Thus, structured learning emerges as the pivotal factor in developing nutritional competency. Therefore, implementing accessible, evidence-based nutrition courses across all university faculties presents a viable and necessary strategy to bridge this persistent knowledge disparity.

We found moderate physical activity was related with reduced odds of unsatisfactory nutrition knowledge in Saudi university students. This pattern has been documented in international studies, where engagement in regular physical activity is often linked to better dietary awareness and healthier eating practices (40–42). In the point of Saudi young adults, this may reflect a growing, multidimensional health consciousness influenced by national public health initiatives, such as the Saudi Vision 2030 health transformation program, which simultaneously promotes physical activity and nutritional awareness (43). Therefore, integrated campus wellness programs must promote physical activity when working with nutrition knowledge improvement.

This study showed that participants with night eating syndrome had a higher probability of possessing unsatisfactory nutrition knowledge. The possible justification is that individuals with disordered eating patterns often exhibit notable gaps in core nutritional information (44, 45). The association is plausibly bidirectional: a deficit in knowledge about meal composition and timing may contribute to dysregulated nocturnal eating, while the night eating syndrome may concurrently impair the acquisition of accurate information or increase exposure to nutritional misinformation. To clarify the direction of this relationship, future longitudinal studies are needed to determine whether poor nutrition knowledge contributes to night eating syndrome development or emerges as a consequence of the disorder, thereby enabling more targeted, causality-informed interventions.

### 4.2 Prevalence and associated factors of health literacy

Our study revealed a high level of limited health literacy, affecting over half (58.3%) of the participating Saudi young adults. This rate is supported by previous research of the general Saudi adult population, which have reported prevalence ranging from approximately 50 to 60% (17–19). Notably, this challenge is not confined to the general population, as even among healthcare students, half report limitations in health literacy knowledge, attitude, and practice (20). The similar prevalence found in this younger, educated cohort suggests that limited health literacy remains a widespread and persistent national challenge. Therefore, targeted health literacy initiatives within universities are critically needed to address this systemic gap in Saudi Arabia.

The finding that participants aged 21–25 years were significantly less likely to have limited health literacy than those aged 18–20 years aligns with international evidence that health literacy often improves with age during young adulthood (46, 47). Since limited health literacy remains highly prevalent overall, the early undergraduate years emerge as a particularly vulnerable period. Therefore, implementing proactive university-wide health literacy interventions, especially tailored for first- and second-year students, is essential to accelerate competency development and reduce this initial disadvantage.

Participants from non-health science majors had an increased probability of demonstrating limited health literacy compared to their health science peers. This disciplinary disparity has been consistently observed across various contexts, where health science students demonstrate superior health literacy due to their direct, curriculum-mandated exposure to health concepts, communication principles, and clinical environments (48). Recent studies, including those in Southeast Asia, confirm that this gap persists and significantly affects how students from different fields access, understand, and apply health information (49, 50). Therefore, universities must implement cross-disciplinary health literacy initiatives to ensure all students, regardless of major, graduate with the essential skills to take decisions regarding their health and healthcare.

Participants with illiterate mothers had higher odds of limited health literacy highlights the significant intergenerational role of maternal education. As primary health socializers, educated mothers are better equipped to foster health-related knowledge and literacy skills at home. This is substantiated by global evidence indicating that lower parental education remains a key social determinant of adverse child outcomes, even after accounting for other socioeconomic factors (51). Therefore, improving national health literacy necessitates family-focused strategies, including adult education and community health programs for mothers, to complement university-level interventions in Saudi Arabia.

### 4.3 Interrelationship between nutrition knowledge and health literacy

The significant reciprocal association observed in our cross-sectional analysis, where limited health literacy and unsatisfactory nutrition knowledge each increased the odds of the other by more than six fold. This indicates a deeply interconnected competency deficit (52). While the direction of causality cannot be established from this study design, the powerful bidirectional relationship aligns with established models in which functional literacy skills are foundational for acquiring and applying domain-specific knowledge (53). This suggests a likely reinforcing cycle, where deficits in accessing and understanding health information hinder nutrition learning, and a lack of nutritional knowledge limits critical engagement with health materials. Future longitudinal or intervention studies are needed to determine the primary direction of this relationship.

### 4.4 Role of nutrition knowledge and health literacy on food consumption patterns

Participants’ food consumption patterns show that vegetables and fruits intake was comparatively low, with only 34.0% of young adults reporting their consumption. This result is corroborated by prior research, which consistently identifies inadequate fruit and vegetable consumption as a public health concern in Saudi Arabia (25). Concurrently, the consumption of unhealthy items was highly prevalent, with a large majority consuming beverages (84.5%), processed items like polysaccharides, canned food, and juices (79.8%), and fast foods (76.3%). This finding aligns with other Saudi studies reporting high frequencies of unhealthy snacking and intake of sugar-sweetened beverages among youth (25, 54). Collectively, these patterns highlight a critical nutritional imbalance and emphasize the dire need for evidence-based initiatives that promote increased intake of protective foods, while reducing the intake of energy-dense, nutrient-poor discretionary items among young individuals in Saudi Arabia.

The present results demonstrate that satisfactory nutrition knowledge is associated with a protective and promotive influence on dietary behaviors, aligning with established theoretical frameworks like the Knowledge-Attitude-Behavior model and reinforcing empirical evidence that links greater nutrition knowledge to higher overall diet quality (55, 56). The significant positive associations between satisfactory knowledge and increased consumption of fruits and vegetables (OR = 1.76) as well as protein-rich foods like meat, fish, and eggs (OR = 2.67) suggest that nutritional literacy facilitates healthier food choices, corroborating previous research which identifies knowledge as a foundational enabler of dietary quality (57) Conversely, the strong association between unsatisfactory knowledge and higher odds of high-calorie food consumption (OR = 3.70) underscores that a knowledge deficit is a key factor for less desirable dietary patterns, consistent with studies linking poor nutritional literacy to increased intake of energy-dense, nutrient-poor foods (25, 58). This relationship is further supported by findings that connect lower nutrition knowledge to adverse health indicators like higher body mass index (55). Collectively, these results highlight a bidirectional relationship where nutrition knowledge serves as both a facilitator of beneficial food consumption and a potential buffer against detrimental dietary practices.

The finding from this study shows that having sufficient health literacy is significantly linked to more frequent consumption of fruits and vegetables. It agrees with the conclusions of (59), who found a strong positive relation between various areas of health literacy and fruit and vegetable intake among college students. They noted that students with excellent health literacy ate significantly more than those with limited literacy. Similarly, the large-scale study by (60) on Chinese college students concluded a clear positive relationship between interactive health literacy and healthier dietary behaviors, including fruit and vegetable consumption, suggesting that higher health literacy promotes better daily dietary choices. Even though our study did not show a significant relationship of health literacy with other food groups, this key finding is conceptually supported by the negative relationship found in other dietary contexts. For instance, a study (61) showed that lower general health literacy was linked to more fast food consumption in an Iranian adult population. This indicates that health literacy serves as a protective factor against poor dietary choices. Consequently, our study’s specific association with fruits and vegetables highlights health literacy as a targeted intervention, as (60) suggested, to improve the important part of diet among young adults.

These findings strongly indicate that both nutrition knowledge and health literacy are key modifiable factors influencing the dietary patterns of young adults. Hence, dual-strategy interventions that simultaneously enhance factual nutrition knowledge and practical health literacy skills are needed to improve diet quality. Specifically, programs should be designed to promote the consumption of fruits, vegetables, and lean proteins while concurrently providing skills to critically evaluate and reduce the intake of refined, nutrient-poor foods.

### 4.5 Limitations of this investigation

First, the design of this work hinders causal inference regarding the identified associations. Second, the use of a convenience sample of university students recruited via an online survey may have introduced potential selection bias. Moreover, this study’s sampling approach narrows the generalizability and applicability of the findings to Saudi Arabia’s young population. Third, as the dietary assessment method did not capture portion sizes or frequency of consumption, it is difficult to draw conclusions about participants’ healthy food consumption patterns. Fourth, the extremely strong reciprocal association between nutrition knowledge and health literacy suggests possible conceptual overlap or shared variance between these constructs, which could not be fully examined in this cross-sectional analysis. Future studies should employ advanced analytical techniques, such as structural equation modeling or factor analysis, to further examined their unique contributions. Lastly, results may be affected by common biases concerning self-reporting, including recall and social desirability bias.

## 5 Conclusion

Our study reported a high level of unsatisfactory nutrition knowledge (70.5%) and limited health literacy (58.3%) among the surveyed young adults (university students). Key factors associated with unsatisfactory nutrition knowledge were enrolled in a non-health science major, physical inactivity, not having taken a nutrition course, night eating syndrome, and limited health literacy. Simultaneously, major factors of limited health literacy were younger age (18–20 years), enrollment in a non-health science major, having an illiterate mother, and unsatisfactory nutrition knowledge. Critically, nutrition knowledge and health literacy demonstrated a reciprocal relationship. Importantly, satisfactory nutrition knowledge and sufficient health literacy were independently related to healthier dietary patterns, including higher consumption frequency of fruits, vegetables, and protein-rich foods. Integrated university-based interventions that simultaneously target health literacy and nutrition knowledge are essential for improving dietary behaviors in this population. Future longitudinal investigation is required to check causal pathways and to evaluate the effectiveness of such integrated programs in promoting sustainable healthy eating patterns and reducing NCD risk among young adults in Saudi Arabia.

## Data Availability

The raw data supporting the conclusions of this article will be made available by the authors, without undue reservation.
